# Impact of preventive therapy on the risk of breast cancer among women with benign breast disease

**DOI:** 10.1016/j.breast.2015.07.013

**Published:** 2015-11-01

**Authors:** Jack Cuzick, Ivana Sestak, Mangesh A. Thorat

**Affiliations:** Centre for Cancer Prevention, Wolfson Institute of Preventive Medicine, Barts & The London School of Medicine and Dentistry, Queen Mary University of London, United Kingdom

**Keywords:** Benign breast disease, Breast cancer prevention, Atypical hyperplasia, Breast cancer risk, Tamoxifen, Aromatase inhibitors, AH, atypical hyperplasia, ADH, atypical ductal hyperplasia, BCRAT, breast cancer risk assessment tool, DCIS, ductal carcinoma *in situ*, HR, hazard ratio, LCIS, lobular carcinoma *in situ*, OR, odds ratio, TDLUs, terminal ductal lobular units

## Abstract

There are three main ways in which women can be identified as being at high risk of breast cancer i) family history of breast and/or ovarian cancer, which includes genetic factors ii) mammographically identified high breast density, and iii) certain types of benign breast disease. The last category is the least common, but in some ways the easiest one for which treatment can be offered, because these women have already entered into the treatment system. The highest risk is seen in women with lobular carcinoma *in situ* (LCIS), but this is very rare. More common is atypical hyperplasia (AH), which carries a 4–5-fold risk of breast cancer as compared to general population. Even more common is hyperplasia of the usual type and carries a roughly two-fold increased risk. Women with aspirated cysts are also at increased risk of subsequent breast cancer.

Tamoxifen has been shown to be particularly effective in preventing subsequent breast cancer in women with AH, with a more than 70% reduction in the P1 trial and a 60% reduction in IBIS-I. The aromatase inhibitors (AIs) also are highly effective for AH and LCIS. There are no published data on the effectiveness of tamoxifen or the AIs for breast cancer prevention in women with hyperplasia of the usual type, or for women with aspirated cysts.

Improving diagnostic consistency, breast cancer risk prediction and education of physicians and patients regarding therapeutic prevention in women with benign breast disease may strengthen breast cancer prevention efforts.

## Benign breast disease and breast cancer risk

It has been known for some time that women with certain types of benign breast disease are at an increased risk of developing breast cancer. A seminal paper was published in 1985 by Dupont and Page [1] who analysed a cohort of 3303 women with histologically confirmed benign breast disease who had been followed up for a median of 17 years. They showed that when compared to non-proliferative lesions, the risk of developing invasive cancer was approximately doubled when the benign lesion demonstrated hyperplasia of the usual type and by 5-fold when atypia was present. This was a major addition to our understanding as previously risk was only clearly associated with lesions showing *in situ* cancer. Ductal carcinoma *in situ* (DCIS) is now considered a precursor lesion as invasive cancer is known to arise directly from it as it is often seen adjacent to invasive cancers and when DCIS is not fully excised invasive cancer often occurs in the same region of the breast. Lobular carcinoma *in situ* (LCIS) however exhibits different properties and is indicative of a generalised abnormality affecting the whole breast. Varying estimates from 2-fold to 13-fold [2], [3], [4], [5] of subsequent cancer risk have been reported, but studies with stringent pathology criteria and long follow up suggest this risk to be 8–10-fold [6], [7]. Subsequent cancer is equally likely to occur in either breast [2].

More recently Hartmann and colleagues [8] have explored the excess risk associated with atypical hyperplasia (AH) in greater detail. Their work indicates that it can be considered as an intermediate endpoint in the cancer process, and when present largely overrides other risk factors such as family history so that the risks are not independent but AH largely dominates and overrides other known risk factors. In particular they found that a family history of breast cancer did not show any modification of the risk associated with diagnosis of AH, in distinction to the original paper by Dupont and Page [1]. They also confirmed this was true in other studies [9], [10], [11]. They have also drawn attention to the importance of involution of breast terminal ductal lobular units (TDLUs) and number of AH foci as important risk-stratifiers. They found that increasing number of foci of AH increase the risk, and complete lobular involution substantially lowers the risk, although it does not reach baseline [10].

Several reports of women with fibroadenoma have also been published [12]. Ciatto et al. [13] found no increased risk when fibroadenoma were not biopsied but only diagnosed clinically (N = 2603, OR = 0.97 (0.70–1.4)) but doubling of risk in those who had a biopsy (N = 1335, OR = 2.00 (1.4–2.7)). This last finding was confirmed by Dupont et al. [14] (OR = 1.61 (1.30–2.0)) who also showed that the risk was greater when either hyperplasia without atypia (OR = 2.16 (1.20–3.8)) or atypical hyperplasia was found (OR = 4.77 (1.50–15)), although the numbers were very small for this last group. McDivitt et al. [15] have furthermore confirmed these findings (Table 1). All these studies were based on excised lesions, where pathologic features could be examined. However, most benign disease is cystic, and is often managed by aspiration to relieve pressure and not excised.

Most studies show little if any excess risk in untreated benign conditions but an increased risk of cancer in women with aspirated cysts has been consistently reported [16], [17]. In particular in a 10 year follow up 1374 women with aspirated but not excised cysts, Dixon and colleagues [18] found a relative risk for developing cancer of 2.81 (2.17–3.59), which is similar to that previously reported by Bundred et al. [19].

## Impact of preventive therapy

### Tamoxifen

Two major prevention trials (NSABP-P1 and IBIS-I) have included women with atypical hyperplasia as a specific entry criterion making them eligible to join these placebo controlled trials of tamoxifen for breast cancer prevention [20], [21]. They comprised 9.1% of the P1 trial and 2.6% of the IBIS-I trial. Both trials also included women with other types of benign disease – the P1 trial accepted women who had 2 or more breast biopsies and the IBIS trial accepted women with excised lesions showing hyperplasia if another risk factor was also present (3.3%).

**Fig. 1:**
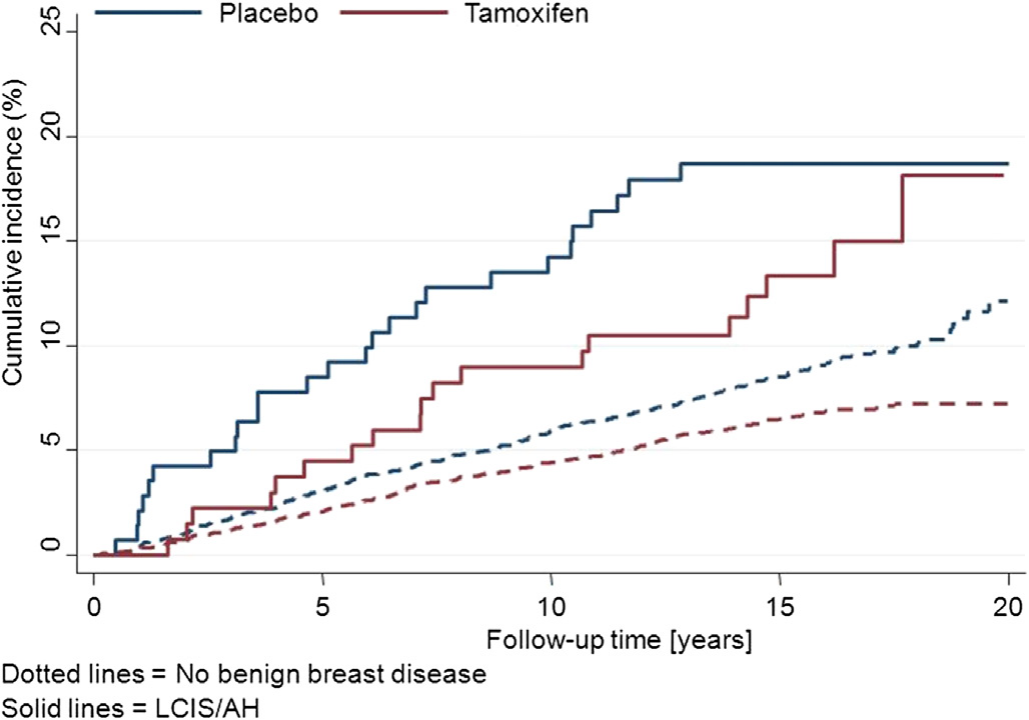
Kaplan–Meier curves for women with LCIS/AH (solid lines) and women without benign breast disease (dashed lines) according to treatment allocation in the IBIS-I trial.

Both trials have reported on the effect of tamoxifen in women with AH or LCIS. In the P1 trial, a larger effect of tamoxifen was seen for women with AH (86% reduction) compared to those in the trial overall (49% reduction) [20]. We have recently reported on the long term follow up of the IBIS-I trial [22] and the results for AH/LCIS vs. no benign disease are shown in Fig. 1 and summarized in Table 2. For women with LCIS at entry, no difference between tamoxifen and placebo was observed (HR = 1.05 (0.48–2.30)). For those with AH, a reduction of 56% was noted for women receiving tamoxifen compared to those on placebo, but the numbers were small and the difference was not significant. The overall reduction for women with a benign breast disease at entry was 29% (HR = 0.71 (0.45–1.12)), which was similar to the overall significant reduction in breast cancer incidence for women without benign breast disease (HR = 0.71 (0.60–0.84)) (Table 2).

**Fig. 2:**
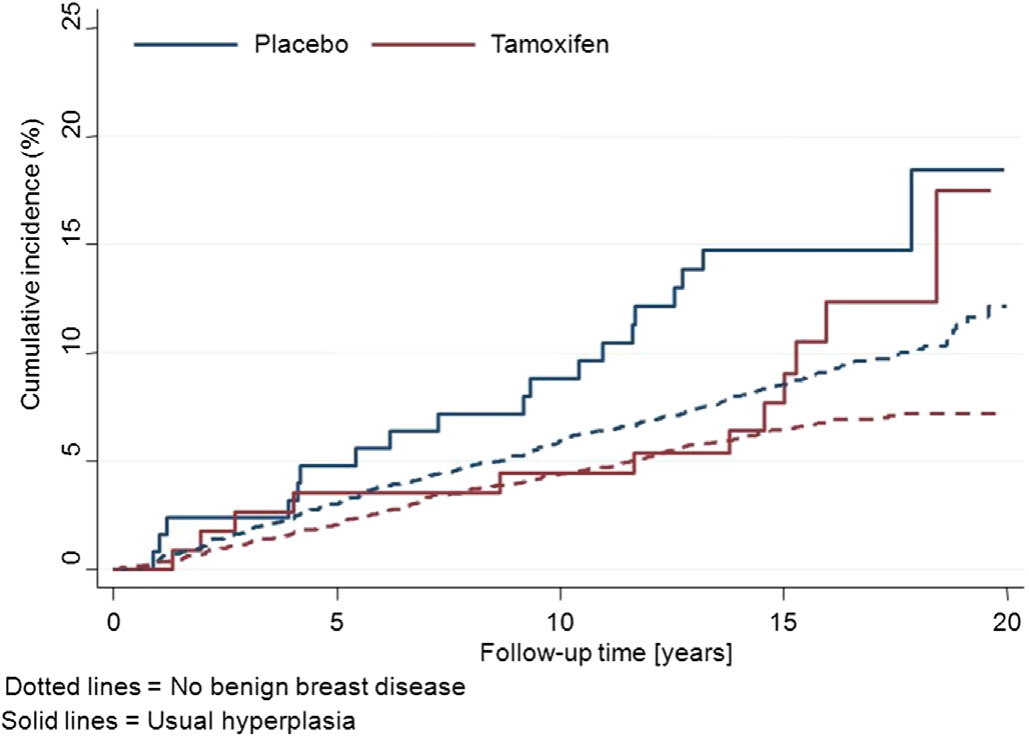
Kaplan–Meier curves for women with hyperplasia without atypia (solid lines) and women without benign breast disease (dashed lines) according to treatment allocation in the IBIS-I trial.

No details are available specifically for women with benign biopsies without histologic evidence of proliferation. Fig. 2 shows the effect of tamoxifen on women with hyperplasia without atypia (and other risk factors) compared to women without proliferative benign lesions. Overall, the 15 year risk was somewhat larger for women with hyperplasia (Table 2) than for women without benign disease (15 year risk in placebo 16.8% vs. 8.5%), but the relative impact of tamoxifen was similar (HR = 0.67 vs. HR = 0.71).

### Aromatase inhibitors

Two trials have evaluated aromatase inhibitors for breast cancer prevention [23], [24], and both have explicitly reported on their effect among women with AH. 8.2% of women in the MAP.3 trial were entered due to LCIS or AH and a reduction in breast cancer incidence of 39% was found with exemestane when compared to placebo (HR = 0.61 (0.20–1.82)). This was smaller than the reduction in those without LCIS or AH, but the difference between two groups was not significant (p = 0.25) [24]. The results for the IBIS-II trial, which used anastrozole are shown in Fig. 1 and Table 3 after a 60-month median follow up. 8.4% of women in the IBIS-II trial were entered due to LCIS or AH. Breast cancer incidence was reduced by 74% with anastrozole for women with LCIS at entry (HR = 0.26 (0.06–1.24)) and a slightly smaller effect was seen for women with AH at entry (HR = 0.35 (0.12–1.07)), which was still larger than that for other women in the trial (Table 3). Although no significant reduction was observed for each individual type of benign breast disease, when combined, a large significant 68% significant reduction (P = 0.01) with anastrozole was seen (HR = 0.32 (0.13–0.79)) (Table 3).

**Fig. 3:**
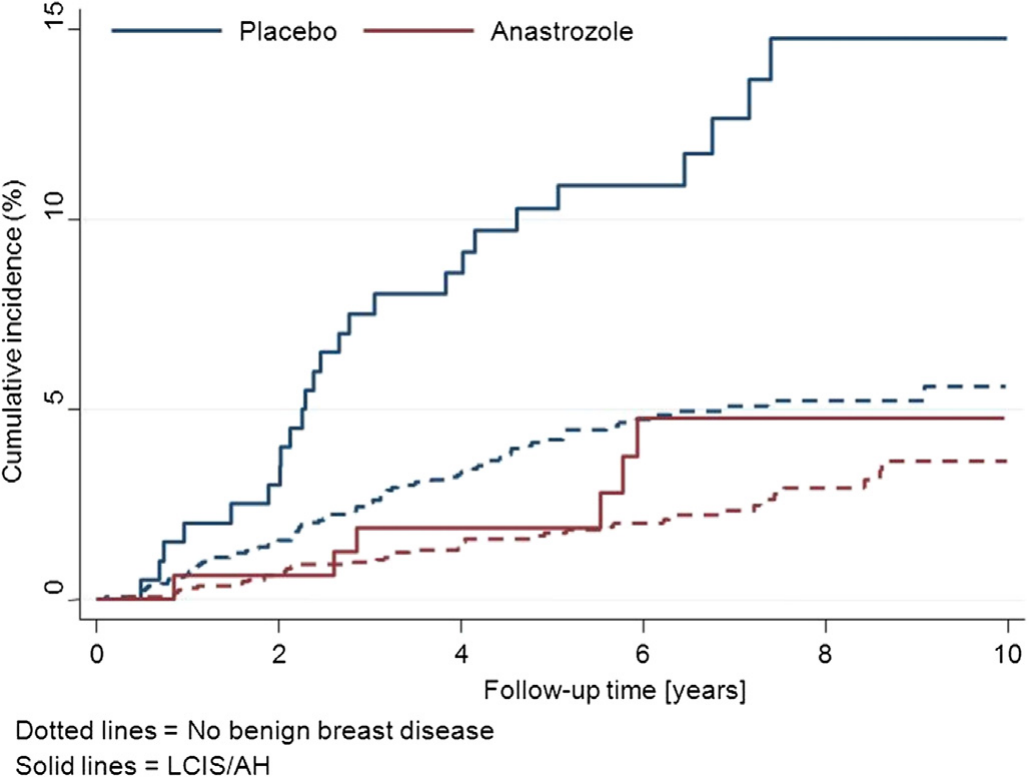
Kaplan–Meier curves for women with LCIS/AH (solid lines) and women without benign breast disease (dashed lines) according to treatment allocation in the IBIS-II trial.

In the IBIS-II trial, women with proliferative disease were also entered on the same basis as for the IBIS-I trial. Here again the risk was higher than for women without benign disease (10 year risk in placebo arms 14.8% vs. 5.6%) and the effect of anastrozole was clearly larger for those with benign breast disease than in those without (HR = 0.29 vs. HR = 0.50; Table 3, Fig. 3). However, there was little difference between LCIS, AH and hyperplasia of the usual type (10 year risks 17.3%, 13.5%, 14.6%, respectively) (Table 3, Fig. 3).

## Challenges in preventive therapy for benign disease and research priorities

### Diagnostic challenges

Considerable inter-observer variation exists for diagnosis of benign breast lesions, particularly for ADH which shares many features of low grade *in situ* DCIS and the distinction between these two lesions remains problematic [25], [26], [27], [28], [29], [30]. Lack of uniformly agreed criteria is one of the reasons for this inconsistency; some base this distinction on size, with lesions smaller than 2 mm being called ADH while others rely on cytological and architectural features. The difficulty in consistently distinguishing between ADH and low grade DCIS is one of the reasons for proposals to classify proliferative ductal lesions in a different manner where both these lesions belong to grade 1 of Ductal intraepithelial neoplasia, albeit different sub-grades [25]. While adoption of such new classification systems can potentially reduce inconsistency, it is unclear whether it will improve breast cancer risk prediction as data for risk stratification based on these new systems do not exist. Improving diagnostic consistency either through addition of biomarkers [28] or adoption of newer classification systems [25] is a research priority; evaluation of newer classification systems through histological reassessment of existing cohorts will be necessary before these are adopted.

### Risk prediction challenges

Commonly used breast cancer risk prediction models, the Breast Cancer Risk Assessment Tool (BCRAT) [31], [32], also known as the Gail model or the International Breast Cancer Intervention Study (IBIS) model, also known as Tyrer–Cuzick model [33] include a component associated with benign breast disease with special categorization of atypical hyperplasia. However further validation of these models when both AH and other important risk factors are present is needed as notable discrepancies have been observed [34], [35]. Accurate risk-prediction is vital for patients and clinicians to determine the absolute benefit of any intervention. Therefore, refinement to improve accuracy of these models and validation of such refined breast cancer risk prediction models is an important research need. Currently, it is worth noting that long term follow up data from Mayo Clinic and Nashville cohorts suggest that AH carries 30% cumulative breast cancer risk over a 25 year period [8].

### Challenges to full implementation of preventive strategies in women with benign disease

A major challenge is full utilisation of preventive therapy in benign breast disease [36]. Physicians, particularly in primary care, are often insufficiently informed about preventive therapy [37] and patients often rely on their physician's recommendation [38]. The decision to not use preventive therapy is often based on an inaccurate assessment of breast cancer risk, i.e. underestimation of disease risk and an exaggerated fear of side effects [38], [39]. As most of this evidence is related to tamoxifen, it is possible that agents with better side-effect profile such as the AIs will be perceived differently. However, better physician education about preventive therapy in breast cancer and availability of improved risk-assessment tools, perhaps with the addition of decision aids during physician-patient discussion, will be essential to overcome these barriers. Development of appropriate decision aids and physician education methods therefore become important research and implementation questions.

## Conclusions

From these results it is clear that women with AH or hyperplasia without atypia are at an increased risk of breast cancer and preventive therapy is particularly effective in this group. Improving diagnostic consistency and breast cancer risk prediction in women with benign breast disease are important research areas. A larger effect on breast cancer prevention for women with benign breast disease was observed with aromatase inhibitors compared to tamoxifen, making this an attractive agent for postmenopausal women with proliferative disease. Furthermore, these women are also an ideal group in which to offer preventive therapy as they have already entered the medical system and have a right to know that they are at an increased risk of developing breast cancer and effective agents exist for reducing that risk. Most women are not aware of these facts and many clinicians are also insufficiently informed about breast cancer preventive therapy. Now that effective strategies for risk reduction have been demonstrated, more effort needs to be expended to make women and their clinicians aware of these possibilities and to provide help in making decisions about preventive therapy.

## Funding

None.

## Author contributions

All authors contributed to writing of the manuscript and approved the final version of the manuscript.

## Disclaimer

The findings and conclusions in this report are those of the authors and do not represent the official position of the authors' respective institutions.

## Conflict of interest statement

JC: Received funding for trials from AstraZeneca. Speakers bureau for AstraZeneca.

All remaining authors have declared no conflicts of interest.

## Figures and Tables

**Table 1:** The risks of subsequent breast cancer for different types of benign disease.

Type	Study (reference)	N (cancer)/N (group)	OR (95% CI)
Atypical hyperplasia (AH)	Nashville [1]	30/232	4.40 (3.10–6.30)
Mayo [8], [10], [11]	143/698	4.34 (3.66–5.12)
Henry Ford [40]	29/246	4.74 (2.81–7.84)
Nurses's health study [41]	96/160^a^	4.11 (2.90–5.83)
McDivitt [15]	66/26^a^	2.60 (1.60–4.10)
Hyperplasia - no atypia	McDivitt [15]	124/68^a^	1.80 (1.30–2.40)
Fibroadenoma (FA) –clinical only	Ciatto [13]	31/2603	0.97 (0.70–1.40)
FA- all histology	Ciatto [13]	41/1335	2.00 (1.40–2.70)
Dupont [14]	87/1835	1.61 (1.30–2.00)
FA – no hyperplasia	Dupont [14]	51/1177	1.48 (1.10–1.90)
McDivitt [15]	64/42^a^	1.70 (1.10–2.50)
FA – hyperplasia without atypia	Dupont [14]	12/162	2.16 (1.20–3.80)
McDivitt [15]	21/6^a^	3.70 (1.50–9.20)
FA – with AH	Dupont [14]	3/19	4.77 (1.50–15.0)
McDivitt [15]	14/2^a^	6.90 (1.50–30.6)
Aspirated cysts – no histology	Dixon [18]	65/1374	2.81 (2.17–3.59)
Bundred [19]	14/352	4.40 (2.41–7.38)

a Case-control.

**Table 2:** Numbers of breast cancer incidence, associated Hazard Ratio (95% CI), and 15-year risk (%) for women with LCIS, AH, hyperplasia, or no-benign breast disease for tamoxifen vs. placebo in the IBIS-I trial.

	Tamoxifen (N = 3579) N (cancer)/N (group)	15 year risk	Placebo (N = 3575) N (cancer)/N (group)	15 year risk	HR (95% CI)
LCIS	13/44	27.0%	12/44	28.0%	1.05 (0.48–2.30)
AH	6/90	6.7%	14/97	14.6%	0.44 (0.17–1.15)
Hyperplasia	12/113	7.7%	19/126	14.7%	0.67 (0.32–1.37)
LCIS or AH	19/134	13.3%	26/141	18.7%	0.73 (0.40–1.32)
LCIS or AH or hyperplasia	31/247	10.8%	45/267	16.8%	0.71 (0.45–1.12)
No benign disease	220/3332	6.5%	305/3308	8.5%	0.71 (0.60–0.84)

**Table 3:** Numbers of breast cancer incidence, associated Hazard Ratio (95% CI), and 10-year risk (%) for women with LCIS, AH, hyperplasia, or no-benign breast disease for anastrozole vs. placebo in the IBIS-II trial.

	Anastrozole (N = 1920) N (cancer)/N (group)	10 year risk	Placebo (N = 1944) N (cancer)/N (group)	10 year risk	HR (95% CI)
LCIS	2/50	4.6%	8/55	17.3%	0.26 (0.06–1.24)
AH	4/98	5.4%	14/123	13.5%	0.35 (0.12–1.07)
Hyperplasia	0/12	0%	3/22	14.6%	0.00 (0.00–2.29)
LCIS or AH	6/148	5.1%	22/178	14.7%	0.32 (0.13–0.79)
LCIS or AH or hyperplasia	6/160	4.8%	25/200	14.8%	0.29 (0.12–0.71)
No benign disease	41/1760	3.6%	80/1744	5.6%	0.50 (0.34–0.73)
